# Multimodal imaging in a case of atypical choroidal osteoma: a 1-year follow-up

**DOI:** 10.22336/rjo.2026.16

**Published:** 2026

**Authors:** Ashish Markan, Nikita Gupta, Ramandeep Singh, Basavaraj Tigari

**Affiliations:** 1 Department of Ophthalmology, All India Institute of Medical Sciences, New Delhi, India; 2 Advanced Eye Center, Post Graduate Institute of Medical Education and Research, Chandigarh, India

**Keywords:** choroidal osteoma, multimodal imaging, FFA, FAF, OCT, OCT-Angio, USG, CNVM = Choroidal Neovascular Membrane, CT = Computed Tomography, EDI-OCT = Enhanced Depth Imaging Optical Coherence Tomography, FAF = Fundus Autofluorescence, FFA = Fundus Fluorescein Angiography, NCCT = Non-Contrast Computed Tomography, OCT = Optical Coherence Tomography, OCT-A = Optical Coherence Tomography Angiography, OD = Oculus Dexter (Right Eye), OS = Oculus Sinister (Left Eye), RPE = Retinal Pigment Epithelium, SS-OCT = Swept-Source Optical Coherence Tomography, USG = Ultrasonography, VEGF = Vascular Endothelial Growth Factor

## Abstract

Choroidal osteomas are rare, benign, ossifying tumors of the choroid that are either flat or slightly elevated, located juxtapapillary, and usually have a well-defined geographic border. We describe a case of choroidal osteoma in a young male with atypical features, including a high elevation of the lesion, blurred margins, and a location away from the optic disc. We also describe the role of multimodal imaging, which allowed us to distinguish an atypical variant of choroidal osteoma from other choroidal mass lesions.

## Background

Choroidal osteoma is a benign tumor of the choroid, where mature bone replaces the choroid [1]. It is considered a choristoma lesion of unknown etiology. Several factors, including inflammation, trauma, hormonal status, genetics, and calcium metabolism, are implicated in its development [2]. Typically, the lesion is flat or slightly elevated and located juxtapapillary, but it may extend into the macula. The lesion has well-defined margins and is yellowish-white, with some peripheral clumping of orange-brown pigments. The ossified tumor can undergo decalcification and show overlying retinal pigment epithelium atrophy. Rarely, a tumor can be associated with choroidal neovascular membranes (CNVM). We describe a case of choroidal osteoma in a young male with atypical location and atypical lesion characteristics.

## Case presentation

**Fig. 1:**
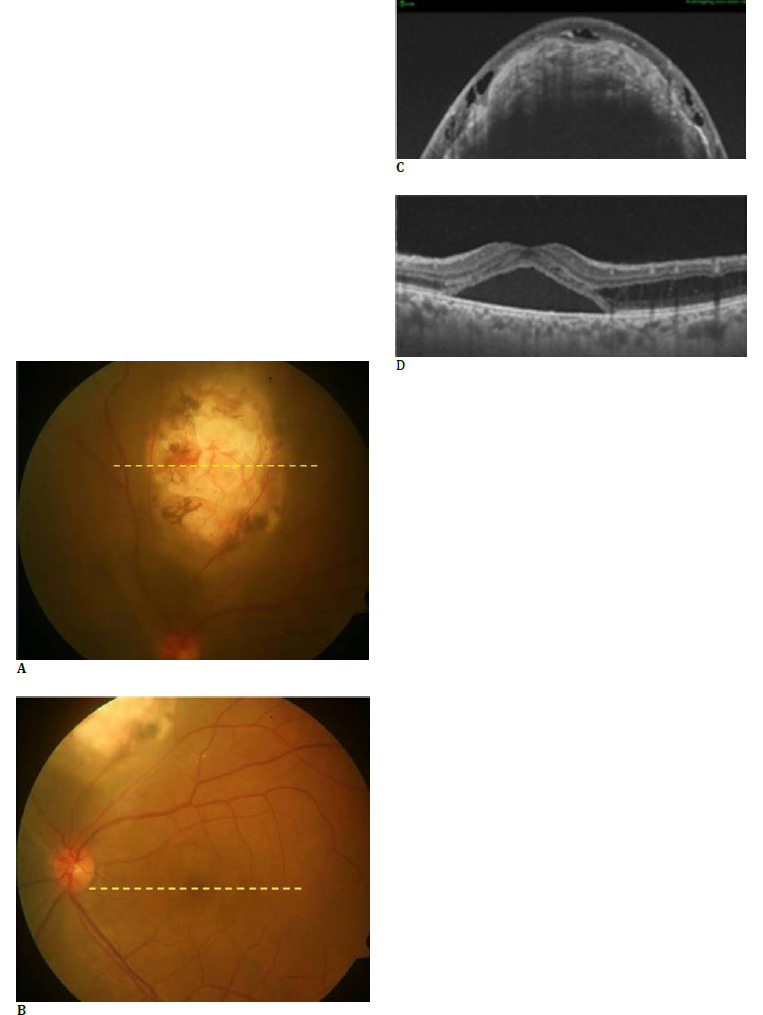
Color fundus photo of the lesion (**A**) showing a yellowish white choroidal mass superior to the disc with pigment clumping and an area of subretinal bleed. Subretinal fluid at the fovea with a dull foveal reflex is also appreciated (**B**). OCT through the lesion (**C**) shows a highly elevated choroidal lesion, with overlying retinal changes. Choroidal architecture showed generalized increased optical density with hyperreflective horizontal lamellar lines and tubules. OCT through the fovea (**D**) shows the presence of subretinal fluid

A 35-year-old male presented with diminution of vision in his left eye (OS) for the last 9 months. There was no antecedent history of ocular trauma. The patient had no known systemic illness. The best corrected visual acuity in the right eye (OD) was 6/6, and in the left eye (OS) was 6/24. Anterior segment examination was unremarkable in both eyes. Fundus examination in the OD was within normal limits. In contrast, fundus examination in the OS revealed a markedly elevated yellowish whitish choroidal lesion (4-5-disc diameters in the greatest linear dimension) superior to the disc (**Fig. 1A, B**). It had blurred margins, a smooth contour, and peripheral black pigmentation. A small area of subretinal bleed was observed in the nasal half of the lesion.

### Investigations

Radial swept-source optical coherence tomography (SS-OCT, DRI OCT-1; Topcon, Tokyo, Japan) passing through the lesion revealed a highly elevated choroidal mass with overlying retinal changes (**Fig. 1C**). The presence of large intraretinal cystic spaces overlying the mass, and disorganization of outer retinal layers with surrounding subretinal fluid was observed. Choroidal architecture showed generalized increased optical density with hyperreflective horizontal lamellar lines and horizontal tubules. The presence of these lamellar lines and tubules within the choroid is usually observed in choroidal osteoma and represents bony lamellae and vascular channels, respectively. Areas corresponding to black hyperpigmentation on clinical examination showed increased signal transmission on SS-OCT owing to loss of overlying RPE with the presence of adjacent hyporeflective spaces in the choroid. OCT scans passing through the fovea showed a localized area of neurosensory detachment with intraretinal splitting along the superotemporal arcade (**Fig. 1D**).

**Fig. 2:**
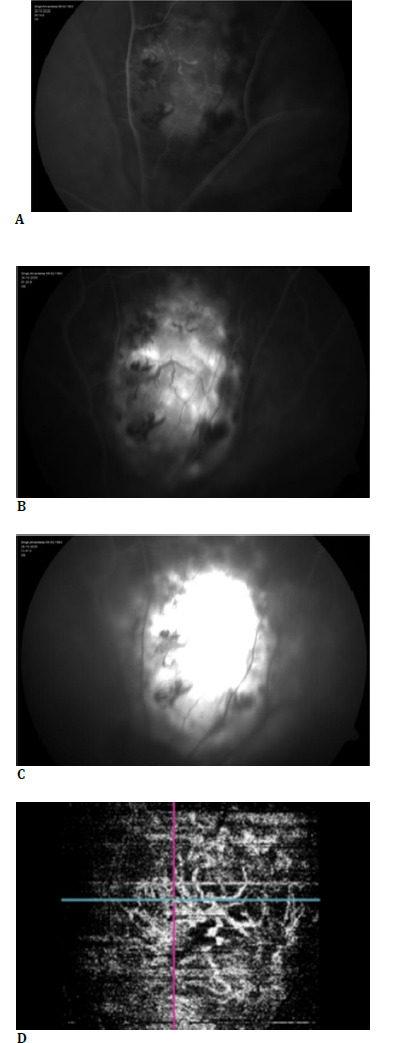
Fundus fluorescein angiography shows early filling of the neovascular complex (**A**). Mottled hyperfluorescence (**B**) followed by late staining of the lesion in the late phase (**C**) was also observed. Optical coherence tomography angiography showed a branching vascular network over the osteoma (**D**)

Fundus fluorescein angiography (FFA) in the early phase showed stippled hyperfluorescence, which progressively increased in the late phase (**Fig. 2A, B**). FFA findings were suggestive of an occult choroidal neovascular membrane. However, late leakage could not be confirmed due to underlying lesion staining (**Fig. 2C**). OCT angiography revealed a neovascular network at the site of the subretinal bleed (**Fig. 2D**).

**Fig. 3:**
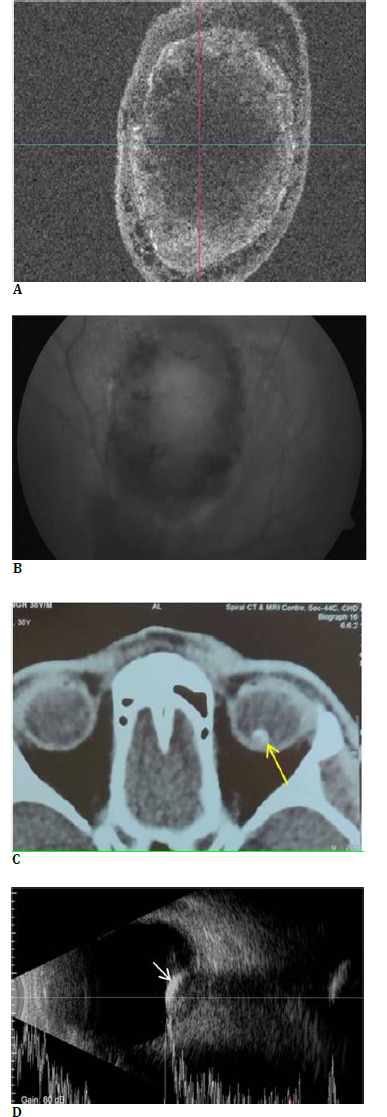
Optical coherence tomography (OCT) enface images (**A**) of the choroidal mass show an oval lesion with a hyperreflective rim and surrounding hyporeflective cystic spaces, giving an eggshell-like appearance. Fundus autofluorescence (**B**) shows central iso-autofluorescence with surrounding hypo-autofluorescence. NCCT scan of the left orbit (**C**) shows a hyperdense rim of calcification (yellow arrow) around the affected choroid. Ultrasonography B/A scan of left globe shows (**D**) a dome-shaped hyperechoic lesion (white arrow) with surface hyperreflectivity and posterior backshadowing suggestive of calcification

Optical coherence tomography (OCT) en face images of the choroidal mass showed an oval lesion with a hyperreflective rim and surrounding hyporeflective cystic spaces, giving an eggshell-like appearance (**Fig. 3A**). Fundus autofluorescence (FAF) showed a central iso-autofluorescent area at the site of the lesion with surrounding hypo-autofluorescence corresponding to the deossified portion of the tumor (**Fig. 3B**).

Non-contrast computed tomography (NCCT) of orbits showed an isodense lesion in the left globe with a peripheral rim of calcification (**Fig. 3C**). Ultrasonography (USG) through the lesion showed a dome-shaped elevation arising from the ocular coats, with an internal hyperechogenicity giving high amplitude spikes and casting an orbital backshadowing (**Fig. 3D**). The above findings were suggestive of calcification within the choroidal lesion.

Based on the above multimodal imaging, a diagnosis of atypical choroidal osteoma with overlying active CNVM was made.

### Outcome and follow-up

**Fig. 4:**
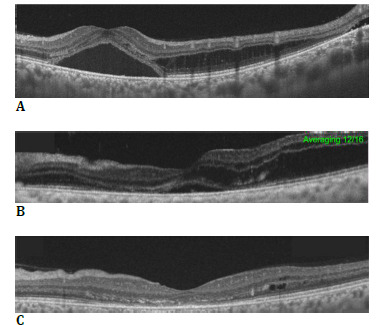
Optical coherence tomography (OCT) images of the patient at (**A**) presentation showing a neurosensory detachment at the fovea, along with intraretinal splitting. (**B**) 9 months after the first visit, OCT showed a marked decrease in the height of the serous detachment, although retinoschisis remained. At 1-year follow-up, (**C**) OCT showed complete resolution of serous detachment and near-total collapse of the retinoschisis cavities without any treatment

The patient was advised to receive an anti-vascular endothelial growth factor injection; however, he did not return for follow-up because of the COVID-19 pandemic at the time. He returned after 9 months with a similar clinical presentation and visual acuity. However, SS-OCT scans through the fovea showed a spontaneous reduction in the height of the serous retinal detachment at the fovea compared with the initial scans (**Fig. 4A, B**). Subsequent follow-up at 1 year showed a near-normal foveal contour, with resolution of retinoschisis superior to the fovea, without any treatment (**Fig. 4C**). The lesion showed no marked change in character, except for an increase in the decalcified area of the tumor.

Although the patient was offered photodynamic therapy for the calcified regions of the lesion to prevent further growth, he declined treatment.

## Discussion

Choroidal osteomas are benign intraocular neoplasms of the eye with mature bone replacing the full thickness of the choroid. They usually occur in young females, with a mean age of 8 to 36 years [1]. However, it has also been reported in males, adults older than 30 years, and children below 10 years [3,4]. They are usually flat or slightly elevated choroidal lesions; however, we could find two case reports of osteoma presenting as elevated lesions, one as high as 5 disc diameters from the retinal surface [5,6]. They are typically observed juxtapapillary or in the macular region. The lesion has a well-defined margin with variable degrees of pigment clumping on the tumor surface. They are usually asymptomatic and are diagnosed in the second or third decade of life, when patients present with decreased visual acuity, visual field defects, or metamorphopsia. The main reasons for the drop in visual acuity are the tumor’s macular location, RPE atrophy over the deossified portion of the tumor, or leakage from the choroidal neovascular membrane associated with the lesion.

Ultrasonography of choroidal osteomas shows high echogenicity of the lesions, which persist at low gains with orbital backshadowing suggestive of calcification. CT scans of the orbit can confirm calcification by showing a hyperdense lesion at the level of the affected choroid. OCT has recently been used to characterize and differentiate choroidal tumors based on the chorioretinal interface and choroidal architecture [7]. EDI-OCT shows a mixed pattern of sponge-like bone and horizontal hyperreflective lamella. Shields et al. classified differences in OCT characteristics between the ossified and deossified portions of the tumor [8]. They showed the calcified portion to be iso- or hyperreflective, with mild signal transmission through the choroid, whereas the deossified portion was usually hyperreflective with increased signal transmission. RPE and photoreceptor atrophy over the decalcified portion of osteoma account for visual loss in these patients. Further characterization of SS-OCT was performed by Azad et al., who showed hyporeflective spaces in the choroid at the junction of ossified and deossified portions, suggestive of bone remodeling via osteoclastic activity [9]. Our case also showed horizontal hyperreflective lamella and hyporeflective spaces in the choroid, suggestive of probable osteoclastic activity. Fluorescein angiography shows mottled hyperfluorescence in the early phase, followed by late staining of the calcified portion of the tumor. Fundus autofluorescence of the calcified untreated region of the tumor shows iso-autofluorescence, while decalcified areas and overlying RPE atrophy show hypo-autofluorescence. Serous retinal detachment shows variable autofluorescence depending on the viability of the overlying RPE [10]. All these ancillary investigations done in our case pointed towards a diagnosis of choroidal osteoma.

Choroidal osteoma needs to be differentiated from other similar lesions, such as amelanotic choroidal melanoma, choroidal metastasis, choroidal hemangioma, choroidal granuloma, or nodular posterior scleritis. In our case, the closest clinical differential diagnosis was amelanotic choroidal melanoma, although it is uncommon in the young population. The tumor typically shows high surface reflectivity on ultrasonography, with low-to-medium internal reflectivity. On OCT, the lesion shows a smooth dome-shaped elevation of the choroid with or without associated exudative retinal detachment. Spontaneous calcification in ocular melanoma is rare, with only 3 cases of intratumoral calcification reported in melanomas without any form of destructive therapy [11-13]. However, this is a slow process, taking years to undergo calcification. Choroidal hemangioma was ruled out because the lesion clinically appears orange-red and shows homogeneous, moderate-to-high internal reflectivity on ultrasonography [14]. Choroidal metastasis can mimic the lesion in our patient; however, such lesions are usually not elevated, are multifocal, and are bilateral in persons with known malignancy elsewhere [15]. Our patient reported no pain, and there were no choroidal folds or disc edema on fundus examination; therefore, we ruled out posterior scleritis.

Shields et al. showed that nearly 50% of choroidal osteomas increase in size over 10 years, and a similar percentage show evidence of decalcification. One-third of the eyes showed the presence of a choroidal neovascular membrane [2]. A long-term follow-up study by Aylward et al. found that the probability of CNVM formation was 47% at 10 years and 56% at 20 years. The likelihood of vision falling below 6/60 was estimated at over 50% within a decade [16]. The disruption of Bruch’s membrane overlying the lesion promotes the growth of neovascular fronds from the choroid.

Various treatment options described for CNVM in choroidal osteoma include laser photocoagulation, transpupillary thermotherapy for extrafoveal tumors, and photodynamic therapy for those close to the fovea [17]. However, the efficacy of these treatments is limited by poor laser uptake due to overlying RPE atrophy. Additionally, laser treatment can promote tumor decalcification, which can further lead to RPE changes and poor vision. Anti-VEGF agents have proven effective by blocking VEGF release induced by ischemia in these slow-growing lesions. The effectiveness of monotherapy with anti-VEGF agents, given a limited number of injections over a long period, has also been reported [18]. Aylward et al. reported spontaneous resolution of serous detachment in approximately 64% of eyes with osteoma over a 10-year follow-up period, with the remaining eyes experiencing poor visual outcomes [16].

This is an atypical case of choroidal osteoma in a young male, as it was neither a flat nor a slightly elevated lesion, as is usually described in the literature. It was a markedly elevated lesion in an atypical location away from the disc. The lesion demonstrated schisis of the inner retinal layers and exudative retinal detachment at the fovea, which resolved spontaneously over one year without treatment. In a community-based case series, Browning et al. reported that 91% of osteomas were misdiagnosed at the initial presentation by ophthalmologists [19]. Although osteomas are slow-growing lesions, a high index of suspicion is warranted to guide correct management and prognosis for the patient. Whenever the diagnosis of a choroidal tumor is uncertain, ultrasonography, OCT, and FFA should be performed to help narrow the differential diagnosis, as in our case.

## Conclusion

This case highlights an atypical presentation of choroidal osteoma characterized by marked lesion elevation, indistinct margins, and a location away from the optic disc. Multimodal imaging, including optical coherence tomography, fundus fluorescein angiography, fundus autofluorescence, ultrasonography, and computed tomography, played a crucial role in establishing the diagnosis and differentiating it from other choroidal mass lesions. The case also demonstrated spontaneous resolution of serous retinal detachment over time without treatment. Careful clinical evaluation combined with multimodal imaging is essential for accurate diagnosis, monitoring disease progression, and guiding appropriate management in patients with atypical choroidal osteoma.

## References

[1] Gass JD (1979). New observations concerning choroidal osteomas. Int Ophthalmol.

[2] Shields CL, Sun H, Demirci H (2005). Factors predictive of tumor growth, tumor decalcification, choroidal neovascularization, and visual outcome in 74 eyes with choroidal osteoma. Arch Ophthalmol.

[3] Behera M, Das MK (2015). A case of choroidal osteoma in a 10-year-old child. Int Med Case Rep J.

[4] Shields CL, Shields JA, Augsburger JJ (1988). Choroidal osteoma. Surv Ophthalmol.

[5] Spies AK, Teitelbaum BA, Aide FK (2001). An atypical case of choroidal osteomas. Optometry.

[6] Cennamo G, Iaccarino G, de Crecchio G (1990). Choroidal osteoma (osseous choristoma): an atypical case. Br J Ophthalmol.

[7] Vishnevskia-Dai V, Zur D, Yaacobi S (2016). Optical Coherence Tomography: An Adjunctive Tool for Differentiating between Choroidal Melanoma and Metastasis. J Ophthalmol.

[8] Shields CL, Perez B, Materin MA (2007). Optical coherence tomography of choroidal osteoma in 22 cases: evidence for photoreceptor atrophy over the decalcified portion of the tumor. Ophthalmology.

[9] Azad SV, Kumar V, Chawla R (2020). In vivo optical biopsy of choroidal osteoma: a swept source optical coherence tomography–based tumor characterization. Ther Adv Ophthalmol.

[10] Sisk RA, Riemann CD, Petersen MR (2013). Fundus autofluorescence findings of choroidal osteoma. Retina.

[11] Chan TK, Atta HR, Scott GB (1995). Ossification in choroidal melanoma. Br J Ophthalmol.

[12] Csákány B, Tóth J (2009). Spontaneous calcification of a choroidal melanoma. Acta Ophthalmologica.

[13] Wang T-W, Liu H-W, Bee Y-S (2019). Distant metastasis in choroidal melanoma with spontaneous corneal perforation and intratumoral calcification: A case report. World J Clin Cases.

[14] Sen M, Honavar SG (2019). Circumscribed choroidal hemangioma: An overview of clinical manifestation, diagnosis and management. Indian Journal of Ophthalmology.

[15] Arepalli S, Kaliki S, Shields CL (2015). Choroidal metastases: origin, features, and therapy. Indian J Ophthalmol.

[16] Aylward GW, Chang TS, Pautler SE (1998). A long-term follow-up of choroidal osteoma. Arch Ophthalmol.

[17] Alameddine RM, Mansour AM, Kahtani E (2014). Review of Choroidal Osteomas. Middle East Afr J Ophthalmol.

[18] Kubota-Taniai M, Oshitari T, Handa M (2011). Long-term success of intravitreal bevacizumab for choroidal neovascularization associated with choroidal osteoma. OPTH.

[19] Browning DJ (2003). Choroidal osteoma: observations from a community setting. Ophthalmology.

